# Unveiling Upper-Limb Dysfunction Early After Implant-Based Breast Reconstruction: Insights from a Retrospective Cohort Study

**DOI:** 10.1007/s00266-026-05915-y

**Published:** 2026-05-26

**Authors:** Elisa Andrenelli, Matteo Torresetti, Enrico Lenti, Ilias Vurliotis, Ortensia Pirro, Maria Gabriella Ceravolo, Giovanni Di Benedetto, Marianna Capecci

**Affiliations:** 1 Department of Experimental and Clinical Medicine, Neurorehabilitation Clinic, Marche Polytechnic University Medical School, Ancona, Italy; 2 Clinic of Plastic and Reconstructive Surgery, Department of Experimental and Clinical Medicine, Marche Polytechnic University Medical School, Ancona, Italy; 3 Breast Surgery Unit, Azienda Ospedaliero Universitaria delle Marche, Ancona, Italy

**Keywords:** Prepectoral breast reconstruction, Functional outcomes, Upper-limb function, Acellular dermal matrix, Mesh, Polyurethane implant

## Abstract

**Background:**

Prepectoral (PP) breast reconstruction has emerged as a muscle-sparing alternative to the subpectoral (SP) approach. While aesthetic and oncologic outcomes have been well studied, early functional recovery remains less explored.

**Methods:**

This retrospective study assessed upper-limb function in women who underwent implant-based breast reconstruction, comparing direct-to-implant PP and two-stage SP approaches. Outcomes at one month included Quick Disabilities of Arm, Shoulder e Hand (QuickDASH) questionnaire, shoulder range of motion (ROM), International Physical Activity Questionnaire (IPAQ), and Body Image Scale. An exploratory analysis within the PP cohort compared outcomes according to implant coverage materials: acellular dermal matrix (ADM), synthetic mesh, and polyurethane-coated (PU).

**Results:**

Among 124 patients, 63 underwent PP and 61 SP reconstruction. PP patients reported lower disability (QuickDASH), better body image, and a lower prevalence of pathological shoulder ROM, particularly in internal rotation and flexion. Functional complications, including adherent scars, dysesthesia, lymphosclerosis, implant hypomobility, and pain, were less frequent in the PP group. Furthermore, fewer PP patients required postoperative rehabilitation. In multivariable analysis, only previous upper-limb dysfunction remained independently associated with higher QuickDASH scores. Within the PP group, ADM-and mesh-based reconstructions were associated with more favorable outcomes than PU implants and remained associated with lower disability after adjustment.

**Conclusion:**

Early postoperative upper-limb function following implant-based reconstruction is influenced by both reconstructive approach and patient-related factors, e.g., preexisting upper-limb dysfunction. Muscle-sparing strategies may support a more favorable functional outcome in the short term, though this hypothesis must be interpreted cautiously given the retrospective design of our study.

**Level of Evidence III:**

This journal requires that authors assign a level of evidence to each article. For a full description of these Evidence-Based Medicine ratings, please refer to the Table of Contents or the online Instructions to Authors  www.springer.com/00266.

## Introduction

Breast reconstruction represents one of the top five reconstructive procedures in the USA, with over 162,000 cases in 2024, according to the American Society of Plastic Surgeons [1]. Postmastectomy implant-based reconstruction has evolved significantly, with increasing adoption of direct-to-implant (DTI) prepectoral (PP) reconstruction alongside the traditional two-stage subpectoral (SP) technique using tissue expanders (TE).

Recent meta-analyses have investigated the safety of PP breast reconstruction in properly selected patients, with all studies demonstrating comparable complication rates between PP and SP reconstruction cohorts [2–4], even in the context of postmastectomy radiation therapy [5].

These findings, combined with the increasing availability of new technologies and ever-evolving techniques, have contributed to the growing adoption of PP reconstruction. Implant coverage with acellular dermal matrix (ADM) and synthetic meshes as interface material, or silicone implants coated with polyurethane (PU), are currently used in clinical practice for PP reconstruction. Consequently, attention has turned to the functional outcomes of PP reconstruction, with several studies addressing this key aspect. While the functional benefits of muscle-sparing reconstruction have already been widely studied [6–8], a focused investigation on early functional outcomes of PP reconstruction based on implant surface type or coating material is still lacking in the literature.

The primary aim of this study was to evaluate early functional outcomes following immediate DTI prepectoral reconstruction compared to the traditional two-step SP reconstruction using a TE, representing two commonly adopted reconstructive pathways in clinical practice. This comparison was chosen as the two-stage approach with TE remains the most commonly performed strategy in implant-based reconstruction, whereas immediate single-stage reconstruction is typically reserved for selected cases [1]. The secondary aims were to evaluate shoulder pain, body image perception, the prevalence of other postoperative functional complications, and the need for rehabilitation in PP and SP groups at one-month follow-up. Finally, we conducted an exploratory subgroup analysis to examine the potential impact of implant surface type and wrapping material on the functional outcomes of inpatients who underwent PP reconstruction.

## Materials and Methods

### Study Design

The research was designed as a retrospective cohort study, conducted following Good Clinical Practice requirements and the 1975 Declaration of Helsinki principles, and submitted to the institutional review board (Comitato Etico Territoriale delle Marche, PREPEC/4252).

### Participants

#### Eligibility Criteria

We enrolled patients undergoing immediate postmastectomy implant-based breast reconstruction at the University Hospital from January 1, 2021, to May 31, 2024, and received physical and rehabilitation medicine consultations one month after surgery.

We identified two patient groups based on the reconstructive technique: The PP group included subjects receiving DTI prepectoral implants, whereas the SP group received a two-stage reconstruction with a TE placed under the pectoralis major muscle. The PP group was further subdivided into three subgroups based on the implant surface/wrapping material: PP-PU group= PP reconstruction with silicone implant coated with PU foam; PP-ADM group = PP reconstruction with textured silicone implant wrapped with ADM; and PP-Mesh group = PP reconstruction with textured silicone implant wrapped with synthetic meshes.

We excluded patients who received breast reconstruction using other techniques (i.e., flap-based breast reconstruction with or without implants) or underwent hybrid reconstruction (i.e., fat grafting with implant).

### Independent Variables

Demographic and clinical data were collected from electronic medical records, including age, comorbidities, smoking status, prior breast surgery, preexisting limitation in upper-limb range of motion (ROM), tumor side, mastectomy type (skin-sparing mastectomy-SSM, nipple-sparing mastectomy-NSM, skin-reducing mastectomy-SRM), surgical indication (oncologic or prophylactic mastectomy), axillary node surgery (sentinel lymph node biopsy- SLNB or axillary lymph node dissection-ALND). Data regarding implant characteristics, including implant type (textured silicone implants or PU-coated implants) and volume, were also collected. Given that, in the SP group, a TE was used and progressive filling occurs over time, the volume recorded at one month does not reflect the final implant volume. Therefore, implant volume was considered a reliable variable only for the PP group.

#### Outcome Measures

We considered early postoperative outcomes at 30 days, including clinical and functional measures, and patient-reported outcomes, including Quick Disabilities of the Arm, Shoulder, and Hand (DASH) questionnaire scores, shoulder ROM on the surgically treated and the contralateral sides, shoulder pain at rest or during motion, upper-limb circumference on the operated and the contralateral side, lymphedema, lymphosclerosis, adherent scars, implant mobility, dysesthesia, Karnofsky disability scale scores, Body Image Scale (BIS) scores, physical activity level (using the International Physical Activity Questionnaire-IPAQ), rehabilitation needs (i.e., physiotherapy sessions for prosthesis mobilization, lymphosclerosis treatment, or shoulder ROM recovery). In cases of bilateral reconstruction, outcomes were analyzed at the patient level, and bilateral procedures were included as a covariate in the multivariable analysis.

### Surgical Procedure

Indications of mastectomy and immediate breast reconstruction were agreed upon by the multidisciplinary breast unit before surgery. The decision to perform SP or PP reconstruction was made intraoperatively based on mastectomy flap thickness and viability. In general, DTI PP reconstruction was selected in patients with adequate soft tissue coverage and well perfused flap, whereas patients with thinner flaps (<1cm) or uncertain perfusion were more likely to undergo a two-stage SP reconstruction with TE placement. The mastectomy flap condition never influenced the choice between ADM/mesh-covered or PU-coated implants, as the reconstructive surgeon predetermined this. In cases of ADM or synthetic mesh use, implants were completely wrapped prior to placement in the prepectoral pocket, ensuring full implant coverage and support. This approach was adopted to optimize implant positioning and stability. The choice between synthetic mesh (TiLOOP® Bra, pfm medical, Cologne, Germany) or acellular bovine pericardium matrix (Exashape, Bioshield Pocket®) was primarily based on institutional availability rather than patient-specific anatomical factors. In the SP group, a TE was placed beneath the pectoralis major muscle.

### Sample Size

The primary endpoint was upper-limb disability, assessed by the Quick DASH scale. A preliminary power analysis indicated that at least 42 patients per group would be required for statistical significance (effect size d = 0.8, significance level alpha = 0.05, beta = 0.2, power 1-beta = 0.8 [80%], 95% confidence interval).

### Statistical Analysis

We described continuous variables using means and standard deviations (SD) or medians and interquartile ranges (IQR), depending on their distribution. We reported categorical variables using absolute values and percentages. We classified a shoulder's active ROM as pathological reduced if the measured values fell below the established cutoff points defined by the confidence intervals (CI) and based on a population of females aged 50 to 59 years [9]. Therefore, the active ROM was considered pathological when flexion was less than 170.5° for the left shoulder or less than 171.8° for the right shoulder; abduction was less than 179.1° for the left shoulder or less than 178.1° for the right shoulder; external rotation was less than 78.8° for the left shoulder or less than 82.2° for the right shoulder; and internal rotation was less than 72.4° for the left shoulder or less than 71.2° for the right shoulder. Shoulder function was therefore evaluated not only through side-to-side comparisons but also against normative cutoff values, allowing identification of pathological limitation independently of the contralateral side, particularly relevant in bilateral cases.

We analyzed differences between PP and SP groups and across PP subgroups (PP-PU, PP-ADM, and PP-Mesh), using the Independent Samples *T*-Test, the Mann-Whitney U Test, or ANOVA/Kruskal-Wallis, depending on the distribution of the variables and the homogeneity of variances, which were assessed using the Shapiro-Wilk test and the Brown-Forsythe test.

Moreover, we used multivariable regression analysis to explore the association between clinically relevant independent variables (including reconstruction plane, previous upper-limb dysfunction, axillary surgery, and bilateral reconstruction in the whole sample, and implant/wrapping material type and implant volume in the prepectoral subgroup) and the Quick DASH scores in the whole sample and in the PP subgroup.

The significance level was set at a p value less than 0.05. Statistical analyses were conducted using JASP software (Version 0.19.1; JASP Team, University of Amsterdam).

## Results

The study included 124 female patients (corresponding to 145 breasts, as 21 subjects underwent bilateral reconstruction) with a mean age of 52.9 years (SD = 8.7): 63 subjects were in the PP group and 61 in the SP group. The descriptive analysis of clinical characteristics and functional outcomes is summarized in Tables 1 and 2.

**Table 1:** Clinical and functional characteristics according to the plane of reconstruction: Prepectoral vs Subpectoral approach

Variables		Prepectoral *N *= 63 (50.8%)	Subpectoral *N *= 61 (49.2%)	P*
Baseline characteristics	Age	52 ± 8.8	53.8 ± 8.6	0.15
	Side reconstruction			0.2
	Left	28	28	
	Right	19	28	
	Bilateral	16	5	
	Previous breast surgery			0.92
	Yes	12	10	
	No	51	46	
	Previous upper-limb limited ROM			0.15
	Yes	5	10	
	No	52	46	
	Type of mastectomy			0.0001
	NSM	58	25	
	SSM	4	35	1
	NSM + SSM	1	1	0.92
	Indication for mastectomy			0.03
	Oncologic	54	60	
	Prophylactic	3	0	
	Oncologic + prophylactic	36	1	
	Node surgery			0.0001
	SLNB	51	29	
	ALND	6	27	
	None	6	5	
Functional measures (early postoperative phase)	Shoulder active ROM on operated side			
	Flexion	146.1 ± 22.5	138.3 ± 22.4	0.06
	Abduction	144.5 ± 26.3	142.9 ± 23	0.43
	External rotation	81.5 ± 11	82.9 ± 10	0.36
	Internal rotation	53.8 ± 18.9	47.3 ± 13.5	0.04
	Shoulder active ROM on contralateral side			
	Flexion	162.3 ± 17.5	161.9 ± 12	0.41
	Abduction	164 ± 15.1	164 ± 11.8	0.64
	External rotation	88.2 ± 4.3	85.5 ± 8.1	0.04
	Internal rotation	56.5 ± 18	52.5 ± 15.2	0.27
	Asymmetry between shoulder ROM (≥20°)			0.0005
	Yes	22	41	
	No	37	18	
	Circumference of operated side			
	Arm	27.3 ± 3.7	27.5 ± 4.7	0.3
	Forearm	22.5 ± 2.7	23.7 ± 2.7	0.02
	Wrist	15.4 ± 1.5	16.2 ± 2.1	0.01
	Circumference of contralateral side			
	Arm	26.9 ± 3.3	27.8 ± 3.3	0.3
	Forearm	22.3 ± 2.7	23.5 ± 2.6	0.06
	Wrist	15.3 ± 1.6	16.3 ± 2.2	0.1
	Circumference difference (operated – contralateral)			
	Arm	0.2 ± 0.9	0.3 ± 1.1	0.9
	Forearm	-0.1 ± 0.8	0.3 ± 1	0.1
	Wrist	-0.01 ± 0.3	-0.03 ± 0.5	0.7
Clinical outcomes	Pain implant/mammary area			
	Yes	30	42	0.035
	No	15	15	
	Shoulder pain at rest	0.7 ± 1.7	0.7 ± 1.6	0.8
	Shoulder pain during active motion	2.1 ± 2.6	2.9 ± 2.8	0.01
	Adherent scar			
	Yes	23	36	0.019
	No	38	25	
	Upper limb dysesthesia			
	Yes	18	38	0.0003
	No	43	43	
	Lymphosclerosis			
	Yes	20	31	0.024
	No	37	24	
	Implant hypomobility			
	Yes	17	41	0.0001
	No	37	40	
Patient-reported outcomes	Karnofsky disability scale	89 ± 6.5	86.2 ± 8.5	0.06
	Quick DASH	22 ± 17.6	32.6 ± 20.2	0.005
	Body image scale-BIS	9.4 ± 7.1	14.9 ± 8.8	0.004
	IPAQ	1643 ± 1111	1608 ± 1287	0.6
Rehabilitation	Postoperative rehabilitation			
	Yes	39	57	0.0001
	No	22	4	

^*^Continuous variables were compared using Student’s t-test or the Mann–Whitney U test, as appropriate; categorical variables were compared using the chi-square test. Side-to-side comparisons should be interpreted with caution in bilateral cases

*ALND* axillary lymph node dissection, *BIS* Body Image Scale, *IPAQ* International Physical Activity Questionnaire, *NSM* nipple-sparing mastectomy, *QuickDASH* Disabilities of the Arm, Shoulder and Hand questionnaire, *ROM* range of motion, *SLNB* sentinel lymph node biopsy, *SSM* skin-sparing mastectomy

**Table 2:** Exploratory analysis of clinical and functional outcomes according to implant/wrapping material in the prepectoral group: silicone implant coated with polyurethane foam (PP-PU group), textured silicone implant wrapped with in acellular dermal matrix (PP-ADM group), and textured silicone implant wrapped with in synthetic meshes (PP-Mesh group)

Variables		PP-ADM *N*=18 (14.5%)	PP-Mesh *N*=22 (17.7%)	PP-PU *N*=23 (18.5%)	p*
Baseline characteristics	Age	50.6 ± 7.3	50.8 ± 10	54.4 ± 8.4	0.37
	Previous upper limited ROM				
	Yes	2	0	2	0.34
	No	16	16	20	
Functional measures (early postoperative phase)	Shoulder active ROM on operated side				
	Flexion	154.7 ± 15.8	145.2 ± 26.2	139.7 ± 22.1	0.107
	Abduction	152.2 ± 10.8	148 ± 31	135.7 ± 28.5	0.119
	External rotation	82.5 ± 7.7	80.2 ± 15.4	81.8 ± 8.2	0.923
	Internal rotation	63.3 ± 17.1	54.2 ± 16.6	45.7 ± 19.3	0.012
	Shoulder active ROM on contralateral side				
	Flexion	173.3 ± 9.6	164 ± 13.3	152.5 ± 20	0.005
	Abduction	169.6 ± 9.4	170 ± 11.5	155 ± 17.2	0.01
	External rotation	88.8 ± 2.3	86.4 ± 6	89.4 ± 3	0.3
	Internal rotation	67.1 ± 15.3	54.6 ± 15	50.3 ± 19.6	0.04
	Asymmetry between shoulder ROM (≥20°)				
	Yes	6	3	7	0.31
	No	12	18	15	
	Circumference of operated side				
	Arm	26.6 ± 2.9	27 ± 4.8	28.4 ± 3.2	0.1
	Forearm	22.1 ± 2.8	22.7 ± 3	22.7 ± 2.5	0.9
	Wrist	15.2 ± 1.4	15.6 ± 1.1	15.4 ± 2	0.5
	Circumference of contralateral side				
	Arm	26.2 ± 2.6	24 ± 2.3	29.1 ± 2.6	0.0004
	Forearm	21.6 ± 2.6	21 ± 2	23.5 ± 2.6	0.03
	Wrist	14.9 ± 1.5	15 ± 0.7	15.8 ± 1.9	0.05
	Circumference difference (operated – contralateral)				
	Arm	0.5 ± 0.8	0.1 ± 0.7	0 ± 1	0.3
	Forearm	0.1 ± 0.8	0 ± 0.3	-0.3 ± 0.9	0.7
	Wrist	0 ± 0.4	0 ± 0	-0.03 ± 0.4	0.8
Clinical outcomes	Pain in implant or mammary region				
	Yes	8	7	15	0.04
	No	9	8	8	
	Shoulder pain during active motion	2.4 ± 2.7	2.2 ± 2.9	1.9 ± 2.3	0.7
	Adherent scar				
	Yes	2	9	12	0.015
	No	16	11	11	
	Lymphosclerosis				
	Yes	6	2	12	0.038
	No	12	14	11	
	Implant hypomobility				
	Yes	0	5	12	0.002
	No	17	9	11	
Patient-reported outcomes	Karnofsky disability scale	90.5 ± 2.3	87 ± 9.2	89.6 ± 5.6	0.29
	Body image scale-BIS	9.4 ± 5.9	7.2 ± 6.1	11.4 ± 8.4	0.27
	IPAQ	2136 ± 940	1583 ± 716	1293 ± 1278	0.009
Rehabilitation	Postoperative rehabilitation				
	Yes	9	12	18	0.16
	No	9	8	5	

*Comparisons across PP subgroups were conducted using one-way ANOVA or the Kruskal–Wallis test, as appropriate. Given the limited sample size within each subgroup, these analyses should be considered exploratory and interpreted with caution. Side-to-side comparisons should be interpreted with caution in bilateral cases

*BIS* Body Image Scale, *IPAQ* International Physical Activity Questionnaire, *QuickDASH* Disabilities of the Arm, Shoulder and Hand questionnaire, *ROM* range of motion

PP and SP groups did not differ in age, history of breast surgery, or previous limited ROM of the upper limb. However, significant differences were observed in mastectomy indication (*p* = 0.033; *χ*^2^=6.857), type of mastectomy (*p *< 0.001; *χ*^2 ^= 37.739), and axillary surgery (*p *< 0.001; *χ*^2 ^= 19.406), with the SP group undergoing more extensive procedures (higher rates of SSM and ALND) compared with the PP group.

At one-month follow-up, patients in the PP group reported less disability and fewer upper extremity symptoms according to the Quick DASH (*p *= 0.005; *U *= 1883.500), higher body image satisfaction without psychological distress (*p *= 0.004; *U *= 1545.500) defined by a cutoff of 11 on the BIS [10].

They also showed lower impairment in shoulder ROM on the operated side, particularly in internal rotation (*p *= 0.048, *U *= 1425.000), with a trend toward reduced limitation in flexion (*p* = 0.06, U=1469.000). Accordingly, pathological shoulder ROM, defined according to normative cutoff values, was more prevalent in the SP group, with higher rates of limited internal rotation (SP: 97% vs PP: 73%, *p* = 0.001; *χ*^2^ = 10.602) and flexion (SP: 97% vs PP: 75%, *p* = 0.002; *χ*^2^ = 9.418).

Significant differences were also observed in the distribution of other functional complications, including adherent mastectomy scars (p=0.019, *χ*^2 ^= 5.547), upper-limb dysesthesia (*p *< 0.001, *χ*^2 ^=  13.203), lymphosclerosis (*p *= 0.024, *χ*^2 ^= 5.109), implant hypomobility (*p *< 0.001, *χ*^2 ^= 25.377), and pain in the implant/mammary area (*p *= 0.035, *χ*^2 ^= 4.466), with a lower prevalence in the PP group. Additionally, the PP group showed a lower need for postoperative rehabilitation treatment (*p *< 0.001, *χ*^2^= 15.837).

Exploratory subgroup analysis based on implant surface/wrapping material (18 patients in the PP-ADM group, 21 patients in the PP-Mesh group, and 23 patients in the PP-PU group) revealed that PP-ADM and PP-Mesh groups exhibited lower Quick DASH scores (*p* = 0.009, *χ*^2^ = 9.420), better shoulder internal rotation (*p* = 0.012, *χ*^2^ = 8.790) and a lower prevalence of pathological internal rotation on operated side defined according to normative cutoff values (PP-ADM: 50%, PP-Mesh: 77.3%, PP-PU: 86.4%, *p *= 0.031; *χ*^2^ = 6.955). Differences were also observed in shoulder ROM on the contralateral side, with significantly lower values in the SP group for flexion (*p* = 0.005), abduction (*p* = 0.01), and internal rotation (*p* = 0.04).

They also exhibited higher IPAQ scores (*p* = 0.009, *χ*^2^ = 9.471), and fewer complications, including mastectomy adherent scars (*p* = 0.015, *χ*^2^ = 8.431), lymphosclerosis (*p* = 0.038, *χ*^2^ = 6.557), and implant hypomobility (*p* = 0.002, *χ*^2^ = 12.439), than the PP-PU group.

Independent factors associated with QuickDASH scores were explored through regression analysis (Table 3). In the overall sample, univariate analysis showed that reconstruction plane and previous upper-limb disability were associated with functional outcomes. However, after adjustment in the multivariable model, only previous upper-limb disability remained significantly associated with higher QuickDASH scores (*p* = 0.007), while the association with reconstruction plane was attenuated and did not reach statistical significance (*p* = 0.072). These findings suggest that differences observed between groups may be partly influenced by baseline clinical factors.

**Table 3:** Factors associated with QuickDASH score at univariate and multivariable analysis (whole sample and PP group)

	Univariate analysis		Multivariable analysis	
	*p*	*t*	*p*	*t*
*PP + SP groups*				
Age	0.692	0.398	0.192	− 1.315
Plane of reconstruction (prepectoral implants)	**0.004**	− 2.920	0.072	-1.824
Previous upper-limb disability	**0.014**	2.492	**0.007**	2.753
Node surgery (sentinel lymph node biopsy)	0.055	− 1.941	0.182	− 1.345
Bilateral reconstruction	0.576	0.560	0.633	0.479
Mastectomy type (NSM)	0.487	− 0.697	0.295	1.054
*PP group*				
Age	0.149	1.464	0.762	-0.305
Implant/wrapping material type polyurethane (Reference)				
ADM	**0.008**	− 2.758	**0.008**	− 2.806
Mesh	**0.009**	− 2.692	**0.016**	− 2.512
Previous upper-limb disability	0.253	1.156	0.646	0.463
Node surgery (sentinel lymph node biopsy)	0.933	− 0.084	0.673	0.425

Significant correlations are indicated by *p* values < 0.05 in bold

*NSM* Nipple sparing mastectomy, *PP* Prepectoral, *SP* Subpectoral

In the PP sample, implant/wrapping material type was significantly associated with QuickDASH scores. Specifically, ADM- and Mesh-wrapped implants were associated with lower disability compared with PU implants (ADM: *p* = 0.008; Mesh: *p* = 0.016). Other variables, including age, previous upper-limb disability, node surgery, bilateral reconstruction, and mastectomy type, were not significantly associated with functional outcomes in the adjusted model.

Within the prepectoral subgroup, implant volume (mean 312.5 cc, SD = 119.7) was further evaluated in an additional multivariable model. Implant volume was not significantly associated with QuickDASH scores (*p* = 0.105; *t*= − 1.658), while the associations observed for ADM- and mesh-wrapped implants remained significant after adjustment (ADM: *p*=0.005, *t *= − 2.945, Mesh: *p *= 0.004, *t *= − 3.010).

## Discussion

The preservation of function, alongside patient well-being and health-related quality of life, represents one of the main goals of breast reconstruction. Nowadays, both patients and clinicians increasingly value a quicker recovery to daily and occupational activities as much as aesthetic outcomes.

Nevertheless, the choice between PP and SP implants after a mastectomy for breast cancer depends on several factors, including the patient’s anatomy, the type of mastectomy, the quality of the remaining tissues, and the preferences of both the surgeon and the patient. In this study, the PP and SP groups did not differ in terms of age or history of breast surgery; however, PP implants were more commonly chosen in cases involving more conservative surgeries (oncological, NSM and SLNB with respect to SSM and ALND). Data recently reviewed from previously published observational studies indicate that there may not be any significant difference in the risk of early surgical complications, such as infection, seroma, hematoma, wound dehiscence, skin necrosis, contracture, or the need for revision surgeries, between the PP and SP groups [11]. However, few studies have specifically focused on functional outcomes. Both techniques have specific advantages and disadvantages [11].

This study suggests that PP reconstruction may be associated with improved functional outcomes in the early postoperative phase. Multivariable analysis showed that the association between reconstruction plane and functional outcomes was attenuated after adjustment, while previous upper-limb dysfunction remained the only independent predictor of postoperative disability. Complications such as scar disorders, lymphosclerosis, or pain at the implant site were less frequent in the PP group, which may contribute to the improved functional outcomes observed in this cohort. Notably, these individuals also showed higher physical activity levels within the first month after surgery, as reflected by the IPAQ score. Eventually, they required less structured rehabilitative treatment compared to the SP group at the one-month follow-up. This finding suggests a more rapid functional recovery in the PP group, with an earlier return to daily activities.

The reconstructive approaches target different anatomical structures, which may result in different functional outcomes and timelines for returning to work and everyday life. The functional impairment associated with SP implant-based breast reconstruction has been thoroughly investigated in the literature. Most studies comparing SP to PP techniques agree that muscle-sparing reconstruction provides better functional outcomes [6–8]. In SP reconstruction, the pectoralis major muscle must be dissected from the chest wall to create the implant pocket, thus reducing pectoralis muscle strength, affecting shoulder flexion, adduction, extension, and internal rotation [12], and eventually leading to shoulder stiffness, instability, and decreased long-term function. Congruently, sparing the pectoralis major muscle through a PP approach would avoid biomechanical alterations [13]. These findings are further supported by recent cadaveric biomechanical evidence demonstrating differences in chest wall dynamics and muscle involvement between PP and SP implant positioning. [14] The role of implant volume should be interpreted with caution, particularly when comparing different reconstructive approaches. In prepectoral reconstruction, implant volume reflects the definitive reconstruction and may influence soft tissue tension and biomechanics. In contrast, in subpectoral reconstruction, tissue expanders are typically used, and the volume at one month does not represent the final implant size, as expansion is performed progressively over time. However, in both two-stage total subpectoral reconstruction and dual-plane DTI reconstruction, the pectoralis major muscle is surgically elevated and partially detached to create the breast pocket regardless of the type of implant used (e.g., tissue expander or prosthesis), which still leads to an alteration of muscle mechanics and a potential compromise of shoulder function [15]. Therefore, early postoperative functional impairment is more likely related to muscle involvement rather than implant volume per se. In this context, the poorer functional outcomes observed in the subpectoral group, despite a lower initial expander volume, further support the role of muscle dissection as a key determinant of early shoulder dysfunction.

The reduced shoulder internal rotation observed in the SP group compared to the PP group supports the hypothesis that pectoralis major muscle disinsertion compromises shoulder function and contribute to the increased need for postoperative rehabilitation treatment observed in the SP group.

Coherently and in line with previous studies [8], SP reconstruction was associated with higher Quick DASH scores than PP reconstruction. Although our functional assessment was conducted early point (one month postoperatively), the data also align with those from Patel et al., who prospectively evaluated patients at 6 months after subpectoral reconstruction and found significantly higher Quick DASH scores [16].

We also found a significantly higher incidence of complications associated with the surgical technique, such as adherent scar, implant hypomobility, and pain in the mammary/implant area. On the other hand, we acknowledge that the increased incidence of lymphoscleroris and upper-limb dysesthesia in the SP group may depend on a greater proportion of women in need of axillary lymph node dissections. Overall, the higher complication rate explains the lower scores on the body image scale in the SP group.

Pain management is another priority of optimizing the breast reconstruction experience. Schaeffer et al. demonstrated better pain control in patients undergoing PP reconstruction compared to those in a case-matched partially submuscular cohort [17]. In line with those results, we observed a higher incidence of pain in the mammary area among SP patients.

The availability of different wrapping materials such as synthetic meshes/ADM or implants coated with polyurethane foam, has driven the increased adoption of the PP approach. Originally introduced to reduce capsular contracture, the ADMs are now used also to camouflage the implant, reduce palpable rippling and implant edge visibility, better tailor the implant pocket stabilizing the prosthesis, and protect the implant in cases of mastectomy flap necrosis or dehiscence. Nevertheless, a recent metanalysis [18] questioned the undisputed benefits of ADM use in PP breast reconstruction, showing no significant differences in infection, capsular contracture, or rippling rates compared to reconstructions without ADM. Reitsamer et al. [19], argued that comparable outcomes may be achieved using synthetic meshes. Immediate PP reconstruction with polyurethane implants is considered a feasible alternative, with outcomes similar to ADM-assisted PP reconstruction, whether used alone or in combination with ADMs [20–22].

In this study subjects in the ADM and Mesh subgroups, compared to those in the PU group, experienced less upper-limb disability, greater mobility of the glenohumeral joint, particularly in internal rotation, and fewer complications, including scar disorders, lymphosclerosis, and implant hypomobility. These differences may be related to the mechanical properties and integration of ADM and synthetic meshes with surrounding tissues which could explain such a positive outcome. Conversely, PU-coated implants may adhere more firmly to surrounding tissues due to their surface characteristics, potentially influencing implant mobility and local biomechanics in the early postoperative phase. This may contribute to reduced shoulder range of motion, although this hypothesis warrants further investigation.

Interestingly, differences in shoulder ROM were also observed on the contralateral side across the prepectoral subgroups, particularly for flexion, abduction, and internal rotation, with the PU group showing lower values compared to ADM- and mesh-based reconstructions. This pattern suggests that the influence of surgical techniques on postoperative function may extend beyond the operated side, involving global adaptations of the shoulder girdle. These findings are consistent with previous evidence indicating that shoulder morbidity after breast cancer treatment can be bilateral, likely due to compensatory movement strategies and altered neuromuscular control [23].

The PP-PU subgroup displayed the highest rate of adherent mastectomy scar and implant hypomobility, likely due to the characteristic adhesiveness of PU implants, and their greater intrinsic rigidity. However, these hypotheses should be further explored in prospective studies with a longer follow-up.

Moreover, patients in the ADM and Mesh groups showed higher levels of physical activity three weeks post-surgery. A quicker return to physical activity is of relevance, considering its critical role in oncologic care. Physical activity improves overall survival rates, enhances physical fitness, reduces cancer treatment-related side effects (such as fatigue and lymphedema), and supports psychological well-being. Regular exercise has also been shown to reduce the risk of cancer recurrence and increase quality of life by improving muscle strength, cardiorespiratory fitness, and mood [24, 25]. McGhee et al. reported that a large percentage of women experienced moderate to severe physical side effects across multiple body regions following breast reconstruction, which negatively impacted both physical function and activity. The highest incidence/severity scores were associated with the following: (i) preexisting physical problems before surgery, (ii) postoperative complications (seroma, infection, necrosis), and (iii) autologous rather than implant-based reconstructions [26].

In our sample, prior upper-limb dysfunction emerged as the only independent predictors of postoperative upper-limb disability. The PP approach was associated with lower disability levels only in the silicone ADM and Mesh-wrapped subgroups. These findings highlight the potential functional benefits of the prepectoral technique and suggest that specific reconstructive implant/wrapping material types may influence postoperative outcomes.

## Study Limitations

The retrospective nature of the study and the short follow-up period (one month) may limit the generalizability of the findings. Functional outcomes assessed at this early time point primarily reflect immediate postoperative recovery and may not capture longer-term functional trajectories or late complications. The lack of preoperative functional assessment prevents evaluation of changes over time and limits causal interpretation of the findings. However, this early assessment may also represent a strength of the study, as it allows evaluation of the initial functional recovery phase, which is highly relevant for patients’ prompt return to daily and working activities. Future studies with extended follow-up are warranted to confirm the persistence of these findings over time.

Nevertheless, real-world data, despite their inherent constraints, are increasingly recognized as valuable for providing insight into clinical outcomes in routine practice and may complement evidence from randomized controlled trials. The intraoperative decision to perform PP or SP reconstruction based on mastectomy flap condition may have introduced selection bias; potentially leading to baseline differences between groups, with patients selected for prepectoral reconstruction presenting more favorable local tissue conditions. Although regression analysis was performed, residual confounding cannot be completely excluded.

The subgroup analysis according to implant/wrapping material type may be limited by the relatively small sample size within each category; therefore, these findings should be considered exploratory and require confirmation in larger prospective studies.

Furthermore, although all surgeons were trained to follow standardized techniques, subtle variations in surgical approaches may have influenced the results.

Finally, the study compared two different reconstructive pathways, namely DTI prepectoral reconstruction and two-stage subpectoral expander-to-implant reconstruction, which may limit the comparability and does not allow isolation of the effect of implant plane alone. However, since the aim was to evaluate early postoperative shoulder function, the comparison may still provide clinically meaningful information on the short-term functional burden associated with commonly adopted reconstructive strategies in routine practice.

## Conclusions

Prepectoral breast reconstruction, particularly when performed using ADM or mesh-wrapped implants, was associated with improved early postoperative functional outcomes and a reduced risk of complications compared to SP reconstruction. These results are clinically relevant, as they reflect the early postoperative phase, during which a faster functional recovery may facilitate a prompt return to daily activities and work. However, these results should be interpreted in light of the study’s retrospective design, potential selection bias, and differences in reconstructive pathways between groups.

Overall, these findings support the importance of incorporating functional considerations into the decision-making process when selecting the most appropriate reconstructive technique for patients undergoing breast cancer surgery. Further prospective and randomized controlled trials are warranted to confirm these findings and explore long-term outcomes.
